# Comparison of surgical outcomes between integrated robotic and conventional laparoscopic surgery for distal gastrectomy: a propensity score matching analysis

**DOI:** 10.1038/s41598-020-57413-z

**Published:** 2020-01-16

**Authors:** Chul Kyu Roh, Seohee Choi, Won Jun Seo, Minah Cho, Yoon Young Choi, Taeil Son, Woo Jin Hyung, Hyoung-Il Kim

**Affiliations:** 1 Department of Surgery, Yonsei University College of Medicine, Seoul, Korea; 2 Gastric Cancer Center, Yonsei Cancer Center, Seoul, Korea; 3 Open NBI Convergence Technology Research Laboratory, Severance Hospital, Yonsei Cancer Center, Yonsei University College of Medicine, Seoul, Korea; 4 Present Address: Department of Surgery, Ajou University School of Medicine, Suwon, Korea; 5 Present Address: Department of Surgery, Korea University College of Medicine, Seoul, Korea

**Keywords:** Gastric cancer, Gastric cancer

## Abstract

This study was aimed to compare the surgical outcomes between conventional laparoscopic distal gastrectomy (CLDG) and integrated robotic distal gastrectomy (IRDG) which used both Single-Site platform and fluorescence image-guided surgery technique simultaneously. Retrospective data of 56 patients who underwent IRDG and 152 patients who underwent CLDG were analyzed. Propensity score matching analysis was performed to control selection bias using age, sex, American Society of Anesthesiologists score, and body mass index. Fifty-one patients were selected for each group. Surgical success was defined as the absence of open conversion, readmission, major complications, positive resection margin, and inadequate lymph node retrieval (<16). Patients characteristics and surgical outcomes of IRDG group were comparable to those of CLDG group, except longer operation time (159.5 vs. 131.7 min; *P* < 0.001), less blood loss (30.7 vs. 73.3 mL; *P* = 0.004), higher number of retrieved lymph nodes (LNs) (50.4 vs. 41.9 LNs; *P* = 0.025), and lower readmission rate (2.0 vs. 15.7%; *P* = 0.031). Surgical success rate was higher in IRDG group compared to CLDG group (98.0 vs. 82.4%; *P* = 0.008). In conclusion, this study found that IRDG provides the benefits of higher number of retrieved LNs, less blood loss, and lower readmission rate compared with CLDG in patients with early gastric cancer.

## Introduction

Laparoscopic distal gastrectomy has been widely used to treat early gastric cancer (EGC)^1^. This procedure provides advantages of minimally invasive surgery (MIS) over open gastrectomy with decreasing postoperative pain, pulmonary complications, hospital stay, and improving quality of life^2–4^. In recent years, robotic surgery has been used as an alternative to laparoscopy for gastrectom y^5^. However, studies have shown that surgical outcomes of robotic gastrectomy were comparable to those of laparoscopic gastrectomy^6–9^.

Conventionally, robotic surgery provides potential benefits over laparoscopic surgery, such as EndoWrist instruments, and an ergonomic console system. In addition to these conventional benefits of robotic surgery, recent robotic surgical systems are equipped with Single-Site platform (Intuitive Surgical, Sunnyvale, CA, USA) and fluorescence imaging technology (Firefly). The Single-Site platform allows to reduce the number of ports during robotic surgery for abdominal organ^10,11^. Fluorescence image-guided surgery shows the possibility of adequate lymphadenectomy through visualization of lymphatic channel and lymph node (LN)^12^. Our institution reported the surgical outcomes of robotic gastrectomy using Single-Site platform^13^ and Firefly technology^14^, however, these technologies have never been applied simultaneously in an integrated procedure.

It was hypothesized that a robotic system integrated with both Single-Site platform and fluorescence imaging technology improve surgical outcomes. Single-Site platform can reduce the number of trocars since one video scope, two robotic arms, and one assistant port are introduced into the abdominal cavity via a single port. This may minimize surgical trauma associated with multiple trocar insertions. Fluorescence imaging can help identify lymphatics during operation. Visualization of lymphatics increase the number of retrieved LNs, which is a surrogate marker for oncological safety, and reduce injury to adjacent pancreatic tissue and vessels during lymphadenectomy.

The aim of study was to compare the short-term surgical outcomes between integrated robotic distal gastrectomy (IRDG) and conventional laparoscopic distal gastrectomy (CLDG). A collective terminology of surgical success was defined to assess the quality of surgery.

## Methods

### Study design and patients

A robotic gastric surgery procedure which uses both Single-Site platform and fluorescence image-guided technology was started at our institution in July 2015. Since then, based on patient preference, both laparoscopic and robotic gastrectomy procedures have been performed. Patients were offered the choice to undergo robotic or laparoscopic gastrectomy. Each patient was given a detailed explanation for each type of surgery and chose the type of surgery before operation. Written informed consent for surgery was obtained from all patients. Between July 2015 and July 2017, 277 consecutive patients underwent IRDG (n = 65) or CLDG (n = 212) gastrectomy. All cases of gastrectomy were performed by a single surgeon (KHI). Patients who underwent total gastrectomy (n = 18), proximal gastrectomy (n = 28), completion total gastrectomy (n = 1), and combined resection (n = 22) were excluded to compare perioperative outcomes of distal gastrectomy. In total, 208 patients were included in our analysis. Among these patients, 56 underwent IRDG while 152 underwent CLDG. The Institutional Review Board of Severance Hospital approved this study and waived the need for written informed consent from the participants (IRB No: 4-2017-0810).

### Integrated robotic distal gastrectomy

In this study, integrated robotic surgery was defined as using of fluorescence imaging technology and Single-Site platform in robotic surgery.

#### Endoscopic indocyanine green injection

**Figure 1:**
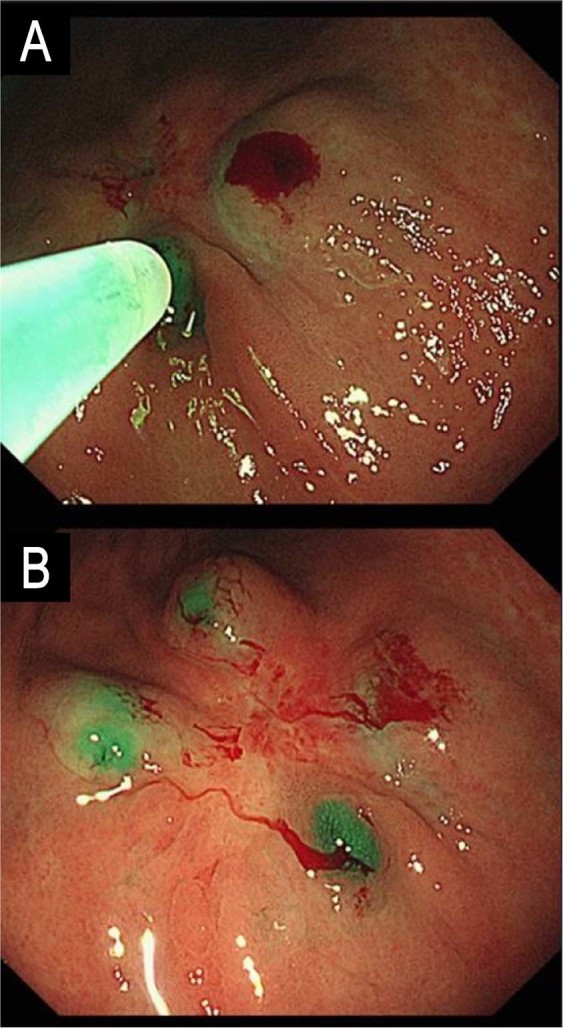
Endoscopic indocyanine green injection. (**A**) Submucosal injection of indocyanine green at the day before surgery (**B**) Post-injection view.

Indocyanine green (ICG, Dongindang Pharmaceutical Co., Siheung, Korea) was used as a near-infrared (NIR) fluorescent contrast agent for intraoperative fluorescence imaging. ICG solution was endoscopically injected into submucosal layer at four sites (0.6 mL at each site, for a total ICG amount of 3 mg) around primary tumor on the day before surgery (Fig. 1), and metal clip was placed for tumor localization.

#### Single-Site platform

**Figure 2:**
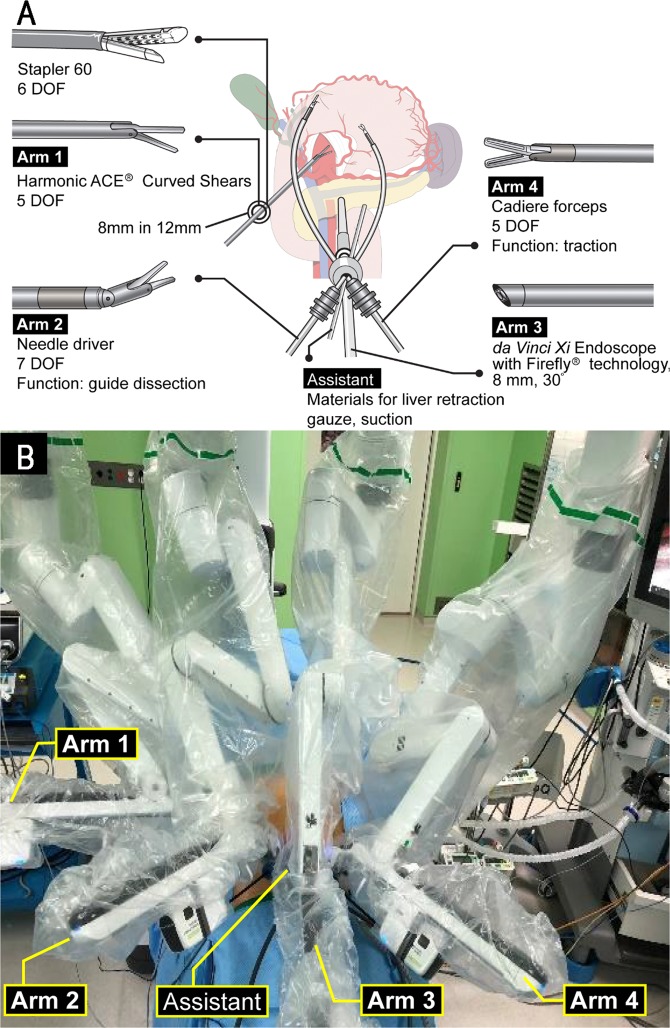
Reduced-port robotic gastrectomy. (**A**) Schematic illustration of reduced-port robotic distal gastrectomy using da Vinci Xi. (**B**) External view after installation Figure 2A is produced by MID (Medical Illustration & Design), a part of the Medical Research Support Services of Yonsei University College of Medicine, which is available under the Creative Commons Attribution 4.0 International License. (https://creativecommons.org/licenses/by/4.0/). DOF, degrees of freedom.

The da Vinci Si or Xi Systems (Intuitive Surgical, Sunnyvale, CA, USA) were used to integrate Single-Site and Firefly technologies. Reduced-port robotic gastrectomy using Single-Site platform for Si system has been described previously^13^. In this paper, surgical procedures are described for Xi systems. Figure 2A illustrates schematic assignment of robot arms for Single-Site. A 12-mm port (XCEL, Ethicon Endo-surgery, Cincinnati, OH, USA) was inserted along the right flank, and an 8-mm straight cannula (470,002, Intuitive Surgical) was inserted in a port-in-port manner docked on the first robotic arm for equipping ultrasonic shears (Harmonic ACE Curved Shears, 480,275, Intuitive Surgical). Single-Site port positioned at umbilical site had four lumens for instruments. Needle Driver (EndoWrist Needle Driver, 478,115, Intuitive Surgical) was inserted through a 5-mm curved cannula (478,072, Intuitive Surgical) docked on the second robotic arm. A 10-mm accessory port (10-mm Accessory Cannula, 428,076, Intuitive Surgical) was inserted to Single-Site port for assistance. The da Vinci Xi 30° Endoscope (470,027, Intuitive Surgical) was inserted through an 8-mm camera cannula (478,063, Intuitive Surgical) docked on the third robotic arm. Cadiere Forceps (EndoWrist Grasper, 478,055, Intuitive Surgical) was inserted through a 5.0-mm curved cannula (478,071, Intuitive Surgical) docked on the fourth robotic arm. Figure 2B shows the external view of robot arms installed for operation.

#### Firefly technology

**Figure 3:**
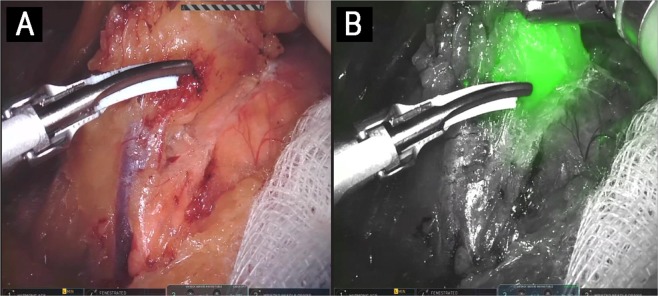
Fluorescence guided lymph node dissection. (**A**) Lymph nodes in white light. (**B**) fluorescence image visualizing lymph node.

The Firefly system was used for fluorescence image-guided lymphadenectomy (Fig. 3). Fluorescence image provides real-time and image-guided identification of lymphatic drainage using NIR technology. Fluorescence imaging improves visual acuity, precision, and control of lymphadenectomy by providing surgeons with a view of lymphatic drainage that is superior to that of the naked eye. D1 imlymphadenectomy (dissection of group D1 and number 8a, 9 LNs) was performed according to the Korean and Japanese gastric cancer treatment guidelines^15,16^.

#### Surgical wound closure after IRDG

**Figure 4:**
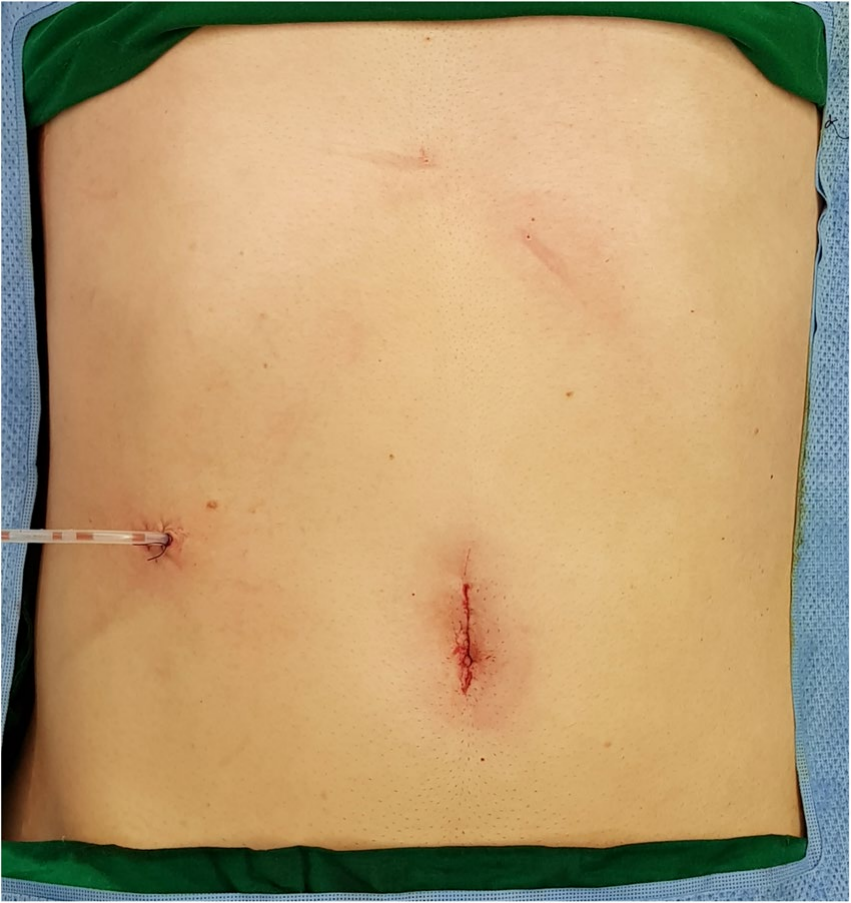
Surgical wound. A drain is inserted via right trocar site. Umbilicus was closed with subcuticular suture.

A closed suction drain was inserted through a 12-mm port site in the right flank. After drain placement, the umbilical wound for Single-Site was closed in layers as follows. First, the peritoneum and fascia layer were closed interruptedly with Polyglactin 910 (VICRYL Plus) 1–0 sutures. Next, for the umbilical wound, subcuticular sutures of both sides of the skin were performed using Polyglactin 910 (VICRYL Plus) 4–0 sutures. Figure 4 photograph shows the umbilical wound and inserted drain after IRDG.

### Conventional laparoscopic distal gastrectomy

The CLDG was performed in standardized surgical procedures as previously described^17,18^. The extent of lymphadenectomy was D1+ as done in IRDG. The reconstruction type was determined according to the location of tumor. Reconstruction method was performed by intracorporeal anastomosis.

### Postoperative management and complication assessment

Both groups received the same postoperative management. Patient-controlled analgesia was used for postoperative pain control until postoperative day 2. Oral analgesics were initiated on postoperative day 3. Using the visual analog scale (VAS), postoperative pain was scored from 0 to 10. Pain score was recorded 30 minutes postoperatively in the post anesthesia care unit (PACU) and recorded at 3, 6, 12, 24, and 48 hours postoperatively in the ward. Sips of water on postoperative day 2 were followed by clear liquid diet on postoperative day 3. Soft diet was allowed on the evening of postoperative day 3. The Clavien-Dindo classification was used to evaluate 30-day postoperative morbidity and mortality^19^.

### Surgical success

In previous studies, it was considered a surgical failure if the following events were observed during the perioperative period^20,21^. (1) conversion to laparoscopic or open surgery for any reasons, (2) harvesting an inadequate number of LNs (defined as <116), (3) positive resection margin, (4) 30-day major postoperative complications defined as grade III or higher according to the Clavien-Dindo classification^19^, or (5) outpatient complications leading to readmission. Any unplanned visit to the emergency department within 90 days after index hospitalization was also regarded as readmission. A low occurrence of these events was considered a prerequisite for successful surgery, ensuring surgical and oncological safety.

### Propensity score matching

Propensity score matching (PSM) analysis was performed to reduce potential selection bias with the following covariates: age, sex, American Society of Anesthesiologists (ASA) score, and body mass index (BMI). Individual propensity scores were calculated using a logistic regression model, and patients between the two groups were matched using the nearest-neighbor matching algorithm (ratio =r1:1 without replacement) with a caliper width of 0.1 standard deviation of the propensity score^22^. After PSM, 102 patients (51 patients each for IRDG and CLDG groups) were analyzed to compare surgical outcomes.

### Statistical analysis

Data were analyzed using IBM SPSS version 23.0 (SPSS Inc., Chicago, IL, USA). Continuous variables were analyzed with paired t test. Chi square test or Fisher’s exact test were performed for categorical variables. Statistical significance was defined as P-value <0.05.

## Results

### Patient demographics

Table 1 lists the clinicopathological characteristics of IRDG and CLDG groups before and after PSM. Before PSM, IRDG group was younger than CLDG group (56.4 vs. 61.8 years; *P* = 0.020). After PSM, matched group showed no significant difference in baseline characteristics.

**Table 1:** Clinicopathological characteristics of patients.

Variable	Entire cohort			Matched cohort		
	IRDG (*n* = 56)	CLDG (*n* = 152)	*P*	IRDG (*n* = 51)	CLDG (*n* = 51)	*P*
**Age**, years	56.4 ± 11.5	61.8 ± 10.7	**0.020**	58.1 ± 10.8	58.0 ± 11.1	0.964
**Sex**			0.234			1.000
Male	28 (50.0)	90 (59.2)		27 (52.9)	27 (52.9)	
Female	28 (50.0)	62 (40.8)		24 (47.1)	24 (47.1)	
**BMI**, kg/m^2^	23.9 ± 2.9	24.1 ± 3.5	0.712	24.0 ± 2.9	24.2 ± 3.7	0.761
**ASA score**			0.073			0.973
1	16 (28.6)	26 (17.1)		14 (27.5)	13 (25.5)	
2	32 (57.1)	86 (56.6)		29 (56.9)	30 (58.8)	
3	8 (14.3)	40 (26.3)		8 (15.7)	8 (15.7)	
**Previous abdominal surgery**			0.173			0.250
No	40 (71.4)	122 (80.3)		36 (70.6)	41 (80.4)	
Yes	16 (28.6)	30 (19.7)		15 (29.4)	10 (19.6)	
pT classification^*^			0.364			0.756
pT1	50 (89.3)	136 (89.5)		46 (90.2)	48 (94.1)	
pT2	2 (3.6)	11 (7.2)		2 (3.9)	1 (2.0)	
pT3	4 (7.1)	4 (2.6)		3 (5.9)	2 (3.9)	
pT4a	0 (0)	1 (0.7)		0 (0)	0 (0)	
**pN classification**^**†**^			0.585			0.517
pN0	51 (91.1)	136 (89.5)		46 (90.2)	49 (96.1)	
pN1	4 (7.1)	9 (5.9)		4 (7.8)	1 (2.0)	
pN2	0 (0)	5 (3.3)		0 (0)	0 (0)	
pN3	1 (1.8)	2 (1.3)		1 (2.0)	1 (2.0)	
**Stage**^**‡**^			0.897			1.000
I	52 (92.8)	139 (91.4)		48 (94.1)	48 (94.1)	
II	3 (5.4)	11 (7.2)		2 (3.9)	2 (3.9)	
III	1 (1.8)	2 (1.3)		1 (2.0)	1 (2.0)	

Data are expressed as mean ± standard deviation or as number (percent).

IRDG, integrated robotic distal gastrectomy; CLDG, conventional laparoscopic distal gastrectomy; BMI, body mass index; ASA, American Society of Anesthesiologists; SD, standard deviation.

^*^pT, depth of invasion.

^†^pN, lymph node involvement.

^**‡**^Stage, according to the 8th edition of the American Joint Committee on Cancer staging system for gastric cancer^32^.

### Surgical outcomes

As shown in Table 2, the mean operative time was longer in IRDG group than in CLDG group (before matching: 157.8 vs. 125.7 min; *P* <0.001, after matching: 159.5 vs. 131.7 min; *P* <0.001). IRDG group was associated with a significantly less estimated blood loss (before matching: 32.4 vs. 68.2 mL; P <0.001, after matching: 30.7 vs. 73.3 mL; *P* = 0.004) and higher mean number of retrieved LNs (before matching: 50.6 vs. 44.6 LNs; *P* = 0.030, after matching: 50.4 vs. 41.9 LNs; *P* = 0.025) than CLDG group.

**Table 2:** Comparison of perioperative surgical outcomes.

Variable	Entire cohort			Matched cohort		
	IRDG (*n* = 56)	CLDG (*n* = 152)	*P*	IRDG (*n* = 51)	CLDG (*n* = 51)	*P*
**Duration of operation**, min	157.8 ± 41.0	125.7 ± 32.6	**<0.001**	159.5 ± 40.6	131.7 ± 33.9	**<0.001**
**Extent of lymphadenectomy**			1.000			1.000
D1+	56 (100)	152 (100)		51 (100)	51 (100)	
**Estimated blood loss**, ml	32.4 ± 32.3	68.2 ± 91.8	**<0.001**	30.7 ± 28.2	73.3 ± 97.1	**0.004**
**Retrieved lymph nodes**, n	50.6 ± 19.2	44.6 ± 16.8	**0.030**	50.4 ± 19.1	41.9 ± 18.5	**0.025**
**Time to first flatus**, days	2.9 ± 0.5	2.9 ± 0.7	0.578	2.9 ± 0.4	2.9 ± 0.7	0.622
**Length of hospital stay**, days	5.0 ± 1.2	5.4 ± 1.9	0.170	5.1 ± 1.2	5.2 ± 0.9	0.781
**CRP**, mg/L						
POD #0	3.2 ± 9.7	1.5 ± 2.7	0.062	3.2 ± 10.1	1.2 ± 2.5	0.165
POD #1	38.3 ± 23.0	30.1 ± 17.6	**0.017**	39.0 ± 21.6	27.2 ± 15.9	**0.002**
POD #3	61.6 ± 38.6	73.4 ± 57.5	0.157	63.5 ± 37.7	65.0 ± 52.5	0.870
POD #4 W	4.8 ± 12.1	5.6 ± 13.1	0.670	5.2 ± 12.6	4.0 ± 7.6	0.564
**Serum Amylase**, U/L						
POD #0	72.5 ± 34.2	67.0 ± 29.5	0.578	71.2 ± 31.7	68.6 ± 27.5	0.659
POD #1	136.9 ± 180.5	136.8 ± 179.2	0.997	138.2 ± 183.7	138.7 ± 198.5	0.990
POD #3	91.4 ± 65.2	86.7 ± 80.8	0.698	88.8 ± 60.0	86.7 ± 55.6	0.848
POD #4 W	84.4 ± 30.8	82.4 ± 26.4	0.646	82.7 ± 30.2	84.1 ± 24.6	0.791
**Serum Lipase**, U/L						
POD #0	31.5 ± 11.5	30.6 ± 11.7	0.641	31.9 ± 11.8	29.4 ± 10.1	0.263
POD #1	33.8 ± 47.0	36.4 ± 68.8	0.793	34.8 ± 49.1	28.1 ± 12.0	0.347
POD #3	39.3 ± 28.0	36.8 ± 55.2	0.738	39.1 ± 28.6	37.9 ± 33.4	0.846
POD #4 W	58.1 ± 35.7	53.7 ± 34.5	0.424	57.5 ± 35.8	56.6 ± 36.6	0.894
**Amount of drainage**, ml						
POD #0	83.0 ± 45.0	113.4 ± 67.0	**0.002**	87.3 ± 44.1	110.1 ± 67.0	**0.045**
POD #1	98.0 ± 86.3	111.0 ± 124.0	0.472	104.3 ± 87.5	122.3 ± 158.2	0.478
POD #2	97.5 ± 83.1	122.4 ± 140.5	0.214	94.8 ± 75.5	136.3 ± 173.1	0.119
POD #3	87.8 ± 80.0	97.1 ± 107.2	0.555	88.4 ± 82.2	101.6 ± 124.0	0.531
**Visual analog scale**						
PACU	4.8 ± 1.5	4.5 ± 1.3	0.173	4.7 ± 1.4	4.4 ± 1.4	0.261
1–3 hours	4.7 ± 1.5	4.6 ± 1.6	0.719	4.8 ± 1.6	5.0 ± 1.6	0.415
3–6 hours	3.7 ± 1.4	3.6 ± 1.3	0.428	3.7 ± 1.3	3.5 ± 1.3	0.408
6–12 hours	3.8 ± 1.4	3.7 ± 1.1	0.702	3.8 ± 1.4	3.7 ± 1.4	0.834
12–24 hours	2.8 ± 1.0	2.7 ± 1.2	0.467	2.8 ± 1.1	2.6 ± 1.2	0.493
24–48 hours	2.1 ± 0.9	1.9 ± 0.7	0.143	2.1 ± 0.9	1.9 ± 0.6	0.124
**Readmission**	1 (1.8)	15 (9.9)	0.075	1 (2.0)	8 (15.7)	**0.031**
**In-hospital complication**			0.387			0.467
G1	19 (33.9)	54 (35.5)		18 (35.3)	16 (31.4)	
G2	6 (10.7)	23 (15.1)		4 (7.8)	8 (15.7)	
G3	0 (0)	3 (2.0)^*^		0 (0)	0 (0)	
**Out-patient complication**			0.848			0.297
G1	0 (0)	1 (0.7)		0 (0)	0 (0)	
G2	0 (0)	3 (2.0)		0 (0)	2 (3.9)	
G3	1 (1.8)^**†**^	5 (3.3)^**‡**^		1 (2.0)	2 (3.9)	
G4	0 (0)	1 (0.7)^**§**^		0 (0)	0 (0)	

Data are expressed as mean ± standard deviation or as number (percent).

IRDG, integrated robotic distal gastrectomy; CLDG, conventional laparoscopic distal gastrectomy; SD, standard deviation; POD, postoperative day; W, week; CRP, C-reactive protein; PACU, post anesthesia care unit.

^*^Intra-abdominal fluid collection, afferent loop syndrome, omental infarction.

^**†**^Internal herniation.

^**‡**^Intra-abdominal fluid collection in two patients, afferent loop syndrome, omental infarction, and internal herniation.

^**§**^Duodenal stump leakage.

Changes in serum levels of C-reactive protein (CRP), amylase, and lipase were examined on postoperative days 0, 1, 3, and postoperative weeks 4. Serum levels of CRP were higher in IRDG group than in CLDG group on postoperative day 1 (before matching: 38.3 vs. 30.1 mg/L; *P* = 0.017, after matching: 39.0 vs. 27.2 mg/L; *P* = 0.002). There were no differences in serum levels of amylase and lipase between the two groups. The amount of drainage was significantly less in IRDG group than in CLDG group on postoperative day 0 (before matching: 83.0 vs. 113.4 mL; *P* = 0.002, after matching: 87.3 vs. 110.1 mL; *P* = 0.045). Visual analog scales at each postoperative period were not statistically different between the two groups.

The readmission rate was significantly lower in IRDG group than in CLDG group (after matching: 98.0 vs. 84.3%; *P* = 0.031). The incidence of in-hospital and out-patient complications were similar between the two groups. There was no operative mortality either group within postoperative 30 days.

### Surgical success

Surgical success rate was significantly higher in IRDG group compared to CLDG group, regardless of matching (Table 3, before matching: 98.2 vs. 89.5%; *P* = 0.046, after matching: 98.0 vs. 82.4%; *P* = 0.008). In IRDG group, surgical failure occurred in only one patient, who was readmitted due to complication identified in outpatient clinic. Except for this one instance, there was no surgical failure in IRDG group regarding major complications, inadequate retrieved LNs (<16), conversion operation, or positive margin.

**Table 3:** Comparison of surgical success.

	Entire cohort			Matched cohort		
	IRDG (*n* = 56)	CLDG (*n* = 152)	*P*	IRDG (*n* = 51)	CLDG (*n* = 51)	*P*
**Surgical success**			**0.046**			**0.008**
**Success**	55 (98.2)	136 (89.5)		50 (98.0)	42 (82.4)	
**Failure**	1 (1.8)	16 (10.5)		1 (2.0)	9 (17.6)	
Readmission	1 (1.8)	15 (9.9)		1 (2.0)	8 (15.7)	
Major complications	1 (1.8)	9 (5.9)		1 (2.0)	3 (5.9)	
Inadequate LN retrieval (<16)	0 (0)	3 (2.0)		0 (0)	2 (3.9)	
Conversion	0 (0)	0 (0)		0 (0)	0 (0)	
Positive resection margin	0 (0)	0 (0)		0 (0)	0 (0)	

Data are expressed as number (percent).

IRDG, integrated robotic distal gastrectomy; CLDG, conventional laparoscopic distal gastrectomy; LN, lymph.

## Discussion

To the best of our knowledge, this study is the first clinical outcome report of robotic surgery integrating Single-Site platform and Firefly technology for reduced-port surgery and fluorescence image-guided lymphadenectomy, respectively. The surgical outcomes following IRDG were notable to those of CLDG in terms of retrieved LNs, estimated blood loss, and readmission, but unfavorable in operation time. Other parameters, including complications, bowel recovery, drainage characteristics, and pain score, did not differ significantly between the two groups. However, regarding the proportion of patients with surgical success, IRDG showed a significantly higher success rate than CLDG.

The advantages of IRDG found in this study were higher number of retrieved LNs and less blood loss compared to CLDG. The number of retrieved LNs is considered a surrogate marker for long-term outcomes and intraoperative bleeding during gastrectomy is known to be related to the risk of tumor recurrence^23–25^. Therefore, the large number of retrieved LNs and low bleeding during surgery suggest that IRDG might be safely used in gastric cancer surgery in terms of oncological aspect. Intraoperative bleeding is also associated with the formation of peritoneal adhesion, which is related to equilibrium disturbance between coagulation and fibrinolysis^26,27^. Even in the absence of previous serosal injury, intraoperative bleeding causes postoperative peritoneal adhesion, a major cause of postoperative complications. However, despite less intraoperative bleeding in robotic gastrectomy, previous studies have shown similar complication rates between conventional robotic and laparoscopic gastrectomy^28,29^. In this study, grade II or higher complication rates were lower in the IRDG group, while it was not statistically significant. Therefore, large-scale prospective study should be performed to confirm that reducing intraoperative blood loss in IRDG may improve postoperative morbidity.

Shortcomings of IRDG found in this study were relatively longer operation time and CRP elevation. Because longer operation time is a common drawback of robot surgery^30,31^, operation time of this study is acceptable. However, CRP level was higher on postoperative day 1 compared to CLDG group. It implies that less invasive surgery through less port site is not effective enough to compensate for longer operation time yet. This study compared the initial experience of IRDG to that of fully optimized CLDG. The learning effect and optimization of IRDG procedures might improve operation time and associated CRP levels.

Surgical success defined in this study was adapted to compare surgical quality following procedures of robot and laparoscopy^20,21^. Since robotic and laparoscopic surgery belong to the same area of minimally invasive surgery, providing surgical benefits that significantly surpass the laparoscopic surgery is a big hurdle for robotic surgery. Thus, collective terminology of surgical success was used to combine the rare unfavorable events in different domains into single parameter. Individual events related with surgical quality, such as complications and inadequate LN retrieval may be insufficient to show significant differences since the number of individual events is very rare. As a combined parameter, surgical success might be effective for comparing the surgical quality of robotic and laparoscopic surgery in this study.

There were several limitations in this study. Due to the retrospective nature of the study, there was a risk of bias in the selection of the patient, even though the patients were matched to balance the two groups.In addition, although IRDG showed some advantages over CLDG in surgical outcomes, which factors, among robot, fluorescence imaging, and reduced-port, contribute to favorable surgical outcomes is still elusive. Therefore, analyzing three factors and comparing them one by one may help determine the contribution of each factor. In addition, there were no results for scar assessment, such as satisfaction for surgical wound or body image. Currently, there has been no study evaluating patient aesthetic satisfaction or quality of life after undergoing reduced-port gastrectomy. Improved aesthetic result or quality of life could be potential benefits of reduced-port gastrectomy. Ongoing prospective study will provide more information on integrated surgery (NCT03396354).

In conclusion, IRDG using the Single-Site platform and fluorescence image-guided lymphadenectomy appears to provide potential benefits in surgical outcomes compared to CLDG for patients with early gastric cancer, relating to retrieved LNs, intraoperative bleeding, readmission, and surgical success.

## Data Availability

The datasets generated during and/or analyzed during the current study are available from the corresponding author on reasonable request.
